# T1 mapping in Cushing's disease: a follow-up study

**DOI:** 10.1186/1532-429X-17-S1-P310

**Published:** 2015-02-03

**Authors:** Roux Charles, Nadjia Kachenoura, Peter Kamenicky, Elie Mousseaux, Philippe Chanson, Alban Redheuil

**Affiliations:** 1 Pierre et Marie Curie Paris VI France, Paris, France; 2 Paris Descartes, Paris, France; 3 Hôpitaux universitaires Paris Sud, Paris, France

## Background

Cushing's disease (CD) has demonstrated subclinical myocardial involvement such as left ventricular (LV) hypertrophy and both diastolic and systolic impairment. Although myocardial fibrosis may play an important role in such myocardial alterations, the new T1 mapping techniques have not yet been used in CD to characterize the myocardium at baseline and to evaluate the effect of treatment on myocardial T1 values.

## Methods

This is a longitudinal pilot study performed in an academic center from Sept 2009 to Jan 2013. Ten consecutive patients (36±15 years, 9 women) with active CD were included and matched with ten healthy volunteers (36±9 years, 9 women) on age, sex and heart rate. Inclusion criteria were: age between 15 and 60 years and newly diagnosed and untreated CD. Subjects had a cardiac MRI exam shortly after diagnosis and a follow-up exam on average 6 month after cortisol normalization. Imaging protocol included MOLLI T1 mapping sequences before and after contrast injection and late gadolinium enhancement (LGE) 10 minutes after contrast injection. Average T1 values from an entire mid-ventricular slice were recorded for each patient before and after treatment and presence of LGE was visually noted.

## Results

While no significant differences were noted between patients and controls in terms of BMI (p=0.08), significantly higher systolic and diastolic pressures were observed in CD (120±14 vs. 100±13, p=0.002 and 75±10 vs. 73±34 mmHg, p=0.023 respectively). No patient had LGE.

Before cortisol normalization, native myocardial T1 was increased in CD patients, as compared to controls (1031±106 ms, 959±45 ms, p=0.049). T1 values 15 minutes after enhancement were not significantly increased. After treatment, native T1 decreased markedly (830±78 ms vs. 1031±106, p=0.004) becoming even lower than controls (p=0.009). T1 value after injection decreased significantly (respectively (p=0.039) in CD patients, reaching values significantly lower than those of controls (p=0.005). These findings favor the hypothesis of differential resolution of edema, interstitial fibrosis induced by CD and steatosis caused by metabolic syndrome which seems to persist.

Before treatment, left ventricular ejection fraction was decreased in patients (p<0.028) and left ventricular mass was increased (p=0.076). After treatment, both parameters tended to normalize without reaching statistical significance.

## Conclusions

The drastic reduction in T1 values after treatment highlight the complexity of myocardial changes and the potential role of other confounders affecting T1 such as water and fat content. T1 after injection need further investigation. Native myocardial T1 is a potential tool to detect myocardial involvement in CD and in its follow-up treatment

## Funding

N/A.

**Figure 1:**
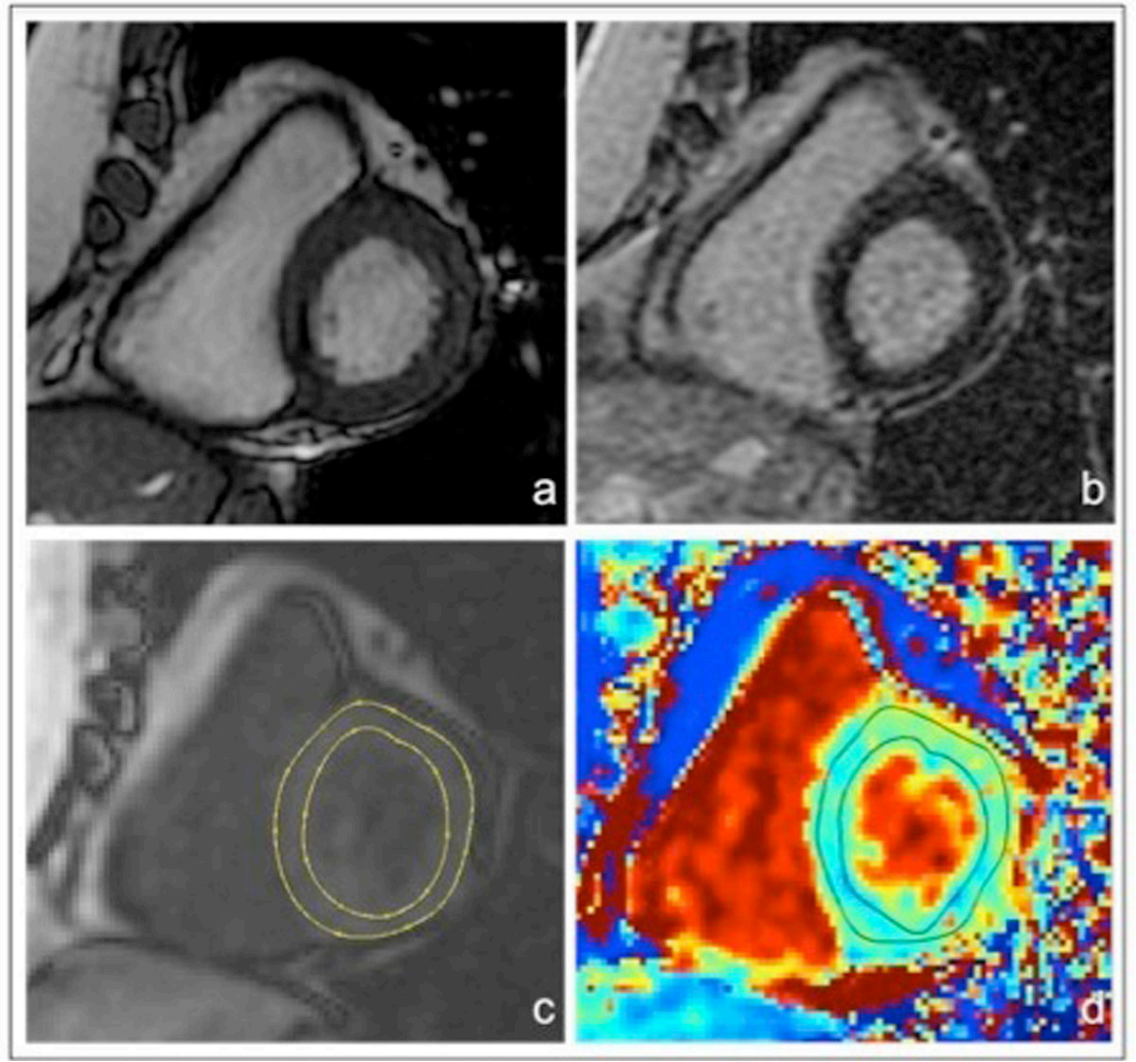
**Myocardial segmentation for native T1 maps, corresponding images.** a) Cine SSFP b) Late gadolinium enhancement c) Epicardial and endocardial borders drawn manually on an original grayscale MOLLI image d) Superimposition T1 map and corrected borders to exclude left ventricular cavity and epicardial fat.

**Figure 2:**
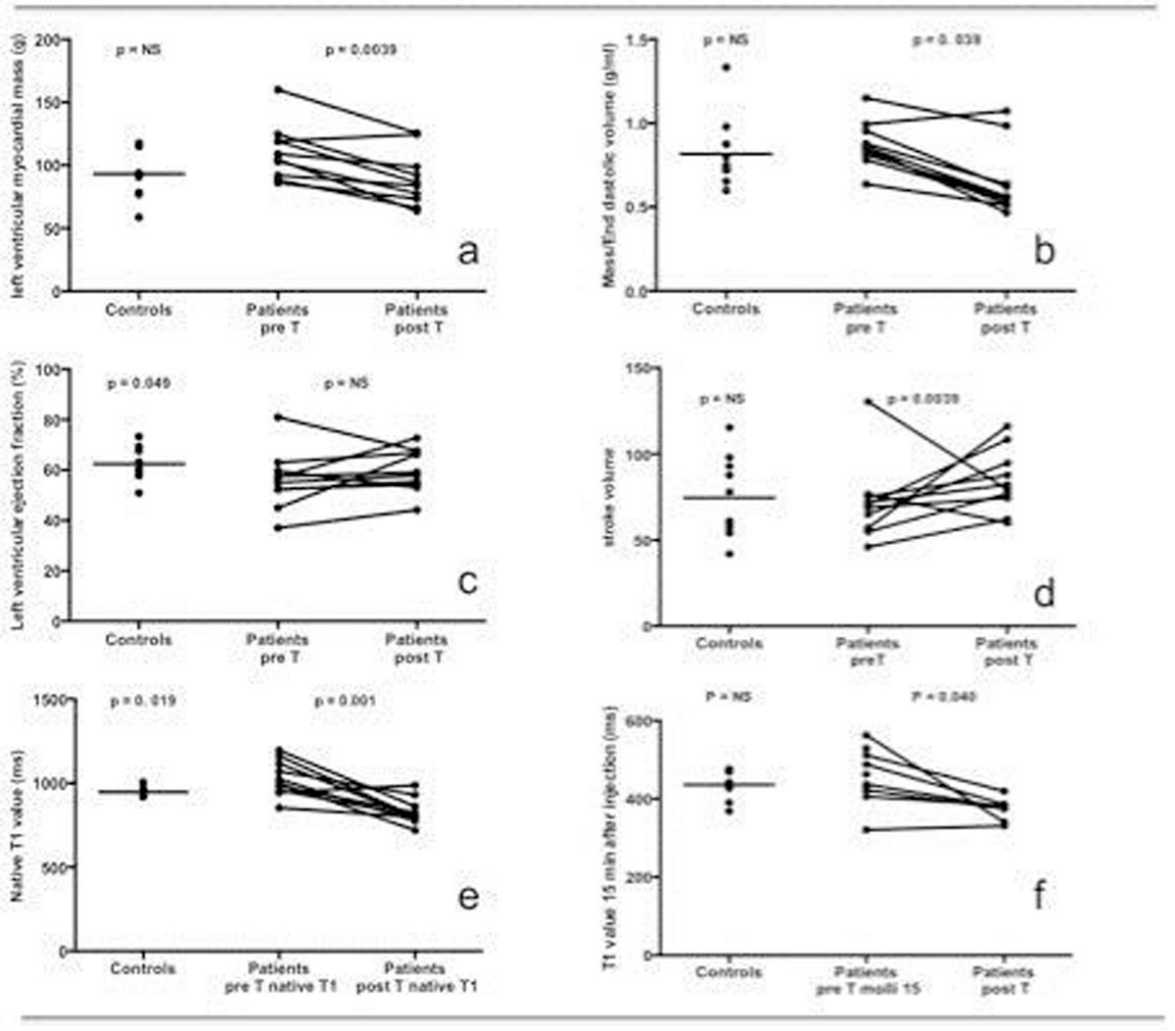
**Comparison of left ventricular myocardial mass** (a), mass/end-diastolic volume (b), left ventricular ejection fraction (c) stroke volume (d), myocardial native T1 value (e) myocardial T1 value 15mn after injection in controls and patients before and after treatment.

